# Knock-in/Knock-out (KIKO) vectors for rapid integration of large DNA sequences, including whole metabolic pathways, onto the *Escherichia coli* chromosome at well-characterised loci

**DOI:** 10.1186/1475-2859-12-60

**Published:** 2013-06-24

**Authors:** Suriana Sabri, Jennifer A Steen, Mareike Bongers, Lars K Nielsen, Claudia E Vickers

**Affiliations:** 1 Australian Institute for Bioengineering and Nanotechnology (AIBN), The University of Queensland, Brisbane, QLD 4072, Australia

**Keywords:** Chromosomal integration, Homologous recombination, Plasmid, Recombineering, *E. coli*, Xylanase, GFP, *csc* genes

## Abstract

**Background:**

Metabolic engineering projects often require integration of multiple genes in order to control the desired phenotype. However, this often requires iterative rounds of engineering because many current insertion approaches are limited by the size of the DNA that can be transferred onto the chromosome. Consequently, construction of highly engineered strains is very time-consuming. A lack of well-characterised insertion loci is also problematic.

**Results:**

A series of knock-in/knock-out (KIKO) vectors was constructed for integration of large DNA sequences onto the *E. coli* chromosome at well-defined loci*.* The KIKO plasmids target three nonessential genes/operons as insertion sites: *arsB* (an arsenite transporter); *lacZ* (β-galactosidase); and *rbsA*-*rbsR* (a ribose metabolism operon). Two homologous ‘arms’ target each insertion locus; insertion is mediated by λ Red recombinase through these arms. Between the arms is a multiple cloning site for the introduction of exogenous sequences and an antibiotic resistance marker (either chloramphenicol or kanamycin) for selection of positive recombinants. The resistance marker can subsequently be removed by flippase-mediated recombination. The insertion cassette is flanked by hairpin loops to isolate it from the effects of external transcription at the integration locus. To characterize each target locus, a xylanase reporter gene (*xynA*) was integrated onto the chromosomes of *E. coli* strains W and K-12 using the KIKO vectors. Expression levels varied between loci, with the *arsB* locus consistently showing the highest level of expression. To demonstrate the simultaneous use of all three loci in one strain, *xynA*, green fluorescent protein (*gfp*) and a sucrose catabolic operon (*cscAKB*) were introduced into *lacZ*, *arsB* and *rbsAR* respectively, and shown to be functional.

**Conclusions:**

The KIKO plasmids are a useful tool for efficient integration of large DNA fragments (including multiple genes and pathways) into *E. coli*. Chromosomal insertion provides stable expression without the need for continuous antibiotic selection. Three non-essential loci have been characterised as insertion loci; combinatorial insertion at all three loci can be performed in one strain. The largest insertion at a single site described here was 5.4 kb; we have used this method in other studies to insert a total of 7.3 kb at one locus and 11.3 kb across two loci. These vectors are particularly useful for integration of multigene cassettes for metabolic engineering applications.

## Background

Integration of large constructs into the *Escherichia coli* chromosome is desirable both for applied engineering projects and for examining metabolic control and other aspects of biological pathways. In particular, metabolic engineering often requires introduction of many genes to achieve production of industrially relevant biochemicals. Whole new pathways may be introduced, as well as genes to increase flux to metabolic precursors, decrease competition from competing pathways, and balance cofactor and redox requirements [1]. The resulting bioprocesses use renewable, sustainable feedstocks such as sucrose and glucose [2,3]; ultimately, lignocellulosic feedstocks will also become available [4]. Recent advances in engineering approaches are allowing for delivery of these ‘green’ replacement products at commercially-viable levels [5].

In *E. coli*, foreign genes may be introduced and expressed from extra-chromosomal elements or integrated onto the host chromosome. While relatively large fragments can be introduced on plasmids, plasmid-based expression has a number of disadvantages. These include the metabolic burden placed on the cell for plasmid maintenance and expression of gene products from multiple copies, the potential for internal rearrangements, segregational instability, fluctuations in copy number and resulting effects on interpretation of data, and the burden placed on cell in presence of antibiotic or other selective agents required for plasmid maintenance [6-11]. Furthermore, unpredictable pleiotropic effects can result from use of plasmids [12], and inclusion of antibiotics for plasmid maintenance increases the overall bioprocess cost. In particular, high copy-number plasmids are known to exert a much stronger metabolic drain than low copy-number vectors [13], and for this and associated reasons, low copy-number vectors are often found to be superior for engineering and other applications [11,14,15].

Integration onto the chromosome solves many of the inherent problems associated with plasmid use, and is therefore a preferable approach in many cases. Constructs can be stably maintained in the absence of selective pressure, the metabolic burden from plasmid maintenance is removed, copy number and segregation are stable and rearrangements are much less likely to occur. Despite the decrease in copy number, productivity can in fact be improved by engineering of genes on the chromosome [16,17].

Chromosomal integration in *E. coli* may be achieved through use of transposons (either random or site-specific; [18,19]) or recombination mediated by phages/phage-derived elements [20-25]. Phages can be used to integrate large DNA fragments. However, the integration sites are fixed, large regions of non-target DNA may be integrated in parallel, and dedicated labs are required for phage manipulation due to the risk of contaminating phage-free strains. Use of phage-derived elements for homologous recombination circumvents several of these problems. For example, specifically-designed integrative plasmids (CRIM plasmids) carrying phage attachment (*attP*) site can be used to insert large DNA fragments at bacterial phage-attachment (*attB*) sites via *in trans* expression of phage-derived integration (*int*) and excision (*xis*) genes [21]. An alternative approach that allows greater flexibility in the insertion locus is the λ Red recombinase system [25-28]. A PCR-based version of this method is widely used for the targeted deletion of chromosomal genes in *E. coli*[23]. This method relies on the replacement of a chromosomal sequence with an antibiotic marker that is amplified by PCR using primers with ~50 bp homology extensions to the flanking regions of the target sequence. The antibiotic resistance gene template is a plasmid; flippase recognition target (FRT) sites are included in order to remove the antibiotic resistance gene after chromosomal integration, allowing recycling of the resistance gene for later engineering steps. Recombination onto the chromosome is mediated by the Red recombinase derived from the λ phage. This recombination system consists of three genes (γ, β, exo), which encode the phage recombinase functions as well as an inhibitor of the host RecBCD exonuclease V (which normally mediates degradation of linear DNA in the cell) [29].

The PCR/λ Red method can also be used for co-integration of relatively short sequences that can be generated through PCR, e.g. introduction of point mutations, promoter replacement, and promoter-less reporter genes for promoter tagging experiments [16,30-34]. However, the efficiency of integration drops sharply for DNA fragments above about ~ 1500 bp and integration of fragments larger than 2500 bp using 50 bp homologous arms is very difficult [23,35], though integration of fragments up to 3,500 bp has been successful [36-38]. Since the resistance gene cassette is 1000–1500 bp (depending on which resistance gene is used), integration of genes of interest is functionally limited to a few genes at a time. This necessitates many iterative rounds of engineering for introduction of large pathways or multiple genes, even when synthetic operons are constructed to minimise sequence length. It has been demonstrated that increasing the region of homology serves to increase the efficiency of integration for large fragments by an order of magnitude [24]. However, this quickly becomes impractical using PCR approaches as the homology region is conferred by the primer sequences, and synthesis of very long primers is both expensive and more error-prone. Furthermore, the frequency of errors in amplified constructs increases with increasing amplicon length, and the yield of amplicons decreases with increasing length.

More recently, two-step techniques that combine the use of the λ Red recombinase with the yeast mitochondrial homing endonuclease I-SceI have been developed for introduction of large DNA fragments onto the *E. coli* chromosome [35,39]. I-SceI has an 18-bp recognition site which is not found in the *E. coli* genome; since double-stranded DNA breaks stimulate *in vivo* recombination, the efficiency of recombination can be increased by several orders of magnitude by using I-SceI sites at the target locus [40]. Two approaches have been described. In the first method, φ80-*attB* sites are introduced onto the chromosome, allowing subsequent integration of a CRIM plasmid bearing *attP* sites and the target DNA fragments (Dual In/Out strategy; [41]). I-SceI sites are used to improve cloning efficiency of large DNA fragments into the CRIM plasmids prior to integration [39]. This approach avoids use of PCR for cloning, and allowed cloning and integration of an 8 kb fragment. The second method involves first using λ Red recombinase to introduce a ‘landing pad’ onto the genome that includes a tetracycline resistance gene flanked by I-SceI sites and short sequences homologous to the ends of the desired integration cassette [35]. Once this landing pad is integrated, the cassette containing the sequences of interest is introduced by digestion with plasmid-expressed I-SceI in the presence of λ Red recombinase. The utility of this method was demonstrated by introduction of a 7 kb fragment at six different loci, and by combinatorial integration at two different loci in one strain. Using these two-step techniques, it is possible to insert very large DNA fragments onto the chromosome in any desired location.

Where flexibility of location is less important than the ability to quickly and efficiently integrate large DNA fragments at well-characterised loci (for example, many metabolic engineering projects), a one-step method of integration is preferable. A second consideration is the lack of availability of well-characterised integration sites for insertion of multiple genes. Insertion at different sites on the chromosome has a significant effect on expression levels; the effect is mediated through interference from local sequences, gene orientation, and insertion position relative to the chromosomal origin of replication [42]. It is therefore of value to know the relative expression levels when genes are inserted at different loci. In addition, it is necessary to avoid disruption of normal growth.

Here we present an alternative, one-step method for integration of large DNA fragments onto the *E. coli* chromosome. The method relies on a series of knock-in/knock-out (KIKO) vectors which facilitate the integration (knock-in) of large DNA sequences onto the *E. coli* chromosome and the parallel interruption (knock-out) of native genes at defined, well-characterised loci using the λ Red system. To increase efficiency of integration, we used large homologous regions (~ 500 bp). Three non-essential loci were characterised as integration sites; combinatorial integration at all three sites in one strain is successfully demonstrated.

## Results and discussion

### Selection of chromosomal sites for recombination

**Figure 1:**
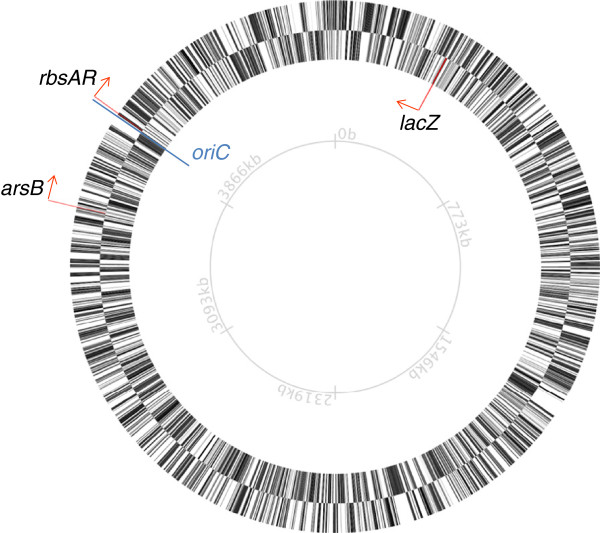
**Location of KIKO target loci on the genome of *E. coli *MG1655.** Map generated using the Microbial Genome Viewer (http://mgv2.cmbi.ru.nl; [47]). Locus positions relative to *oriC* are conserved in *E. coli* W. Arrows represent gene orientation.

The target loci used for development of the KIKO vectors were selected with laboratory/industrial growth in mind. Two of the loci are involved in PTS uptake of compounds typically not used in laboratory or industrial growth conditions: the arsenite transporter *arsB*[43], and the ribose transporter operon *rbsA*-*rbsR*[44]. The third locus was the well-characterised lactose catabolic enzyme gene β-galactosidase, *lacZ*. It has been previously demonstrated that interruption of these ORFs is not deleterious for *E. coli* growth or cell division under standard cultivation conditions [43,45]. To ensure broader application to different *E. coli* strains, these loci were also screened and found to be conserved across a large cross-section of *E. coli* strains, including B, C, W, and K-12 as well as many pathogenic strains (data not shown). The *rbs* operon (*rbsABCKR*) is incomplete and non-functional in *E. coli* W, an industrially relevant sucrose utilizing strain [2], however the target sequences for homologous recombination are conserved. The *lacZ* locus is often subjected to modification in laboratory strains, and has been successfully used as a site for integration of sucrose utilization genes in *E. coli* K-12, C and B [46]. Furthermore, when using *lacZ* as a target locus, blue/white screening can be used to facilitate identification of insertion mutants. The positions of all three loci on the *E. coli* MG1655 genome are shown in Figure 1.

### Design and construction of the KIKO vectors

**Figure 2:**
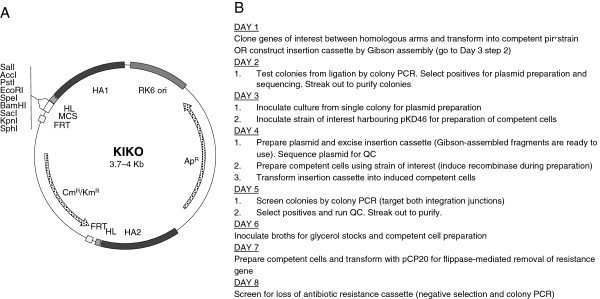
**Generic features of the KIKO plasmids and integration protocol. A**) Map of the general features of the KIKO vectors, showing the location of the antibiotic resistance cassettes (Ap^R^, ampicillan resistance; Km^R^, kanamycin resistance; Cm^R^, chloramphenicol resistance), the flippase recombinase target sites (FRT), the hairpin loops (HL), the homologous arms (HA1 and HA2) used for recombination onto the *E. coli* chromosome and the unique restriction sites located within the multiple cloning site (MCS). **B**) Process for the isolation of integration mutants. This represents the fastest possible generation protocol by standard cloning; in practice, strain construction is often delayed be a few days for quality control (QC). Strain construction can be shortened by two days if insertion cassettes are constructed by Gibson assembly.

The generic features of the KIKO vectors are shown in Figure 2A. They share a common backbone encoding the R6K *pir*^+^-dependent origin of replication (*ori*) and an ampicillin antibiotic resistance cassette. The R6K *ori* requires the π protein, which is supplied in *trans* by the λ*pir* gene [48,49]. Use of the R6K *ori* ensures that the plasmid cannot be maintained in target strains lacking λ*pir*; therefore, antibiotic resistance should not occur in the absence of integration. Homologous arms of ~500 bp each for each target locus are included in the KIKO vectors; this large amount of homology serves to increase recombination efficiency [24]. Embedded between the homologous arms is a multiple cloning site (to facilitate the insertion of genes of interest) as well as an antibiotic resistance gene cassette (chloramphenicol or kanamycin). The antibiotic resistance cassette is flanked by flippase recombination target (FRT) sites to allow for the removal of the antibiotic resistance cassette after insertion of the construct onto the chromosome so that these markers maybe reused as necessary [50,51]. There are three main influencers on gene expression for inserted DNA: local sequence context, gene location, and orientation [42]. Of these, the effect of local sequence context is the most pronounced when expression is controlled by constitutive promoters and the use of transcriptional terminators is absent [42]. Therefore, the entire insertion construct was flanked with sequences encoding hairpin loops [52] to act as transcriptional terminators. These repeats effectively isolate the integrated genes from the influence of transcriptional events at adjacent loci [42]. The process for one round of integration using these vectors is shown in Figure 2B.

### Integration at the target loci does not affect growth rate

**Figure 3:**
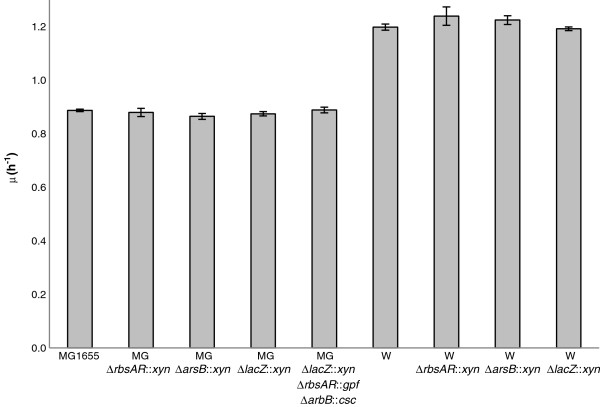
**Specific growth rate (μ; hr**^**-1**^**) of engineered strains.** The growth rate of *E. coli* K-12 (MG1655) and W strains harbouring exogenous DNA integrated into various different loci was measured on LB medium in the absence of selection to allow comparison with wild type strains. Bars are means; errors are standard deviations (*n* = 3). No significant differences were identified for either strain (1-way ANOVA; p>0.05).

We tested the KIKO vectors using two different *E. coli* strains: K-12 (MG1655) and W. *E. coli* K-12 (MG1655) and its derivatives are widely used in both laboratory and industry [53-55] and chromosomal integration using the λ Red functions is routine in this strain. *E. coli* W is of interest as it is particularly fast-growing on sucrose (1.2 h^-1^; [56]), an especially attractive renewable carbon source for industrial applications [2]. *E. coli* W also demonstrates low acetate production enabling cultivation to high cell densities and high tolerance to environmental stresses (reviewed in [2]). To examine the effect of integration at the three selected loci, a FRT:xylanase:FRT cassette was inserted at each site in both W and K12 using the λ Red recombination system [23]. Integration did not affect growth rate in either strain for any of the loci (Figure 3), indicating that the integration of exogenous DNA at these loci is not deleterious for growth rate or viability under standard laboratory conditions. As we have previously observed [46], strain W has a relatively high growth rate; in this case, on LB medium, W grows at 1.2 hr^-1^ compared to 0.9 hr^-1^ for K-12(MG1655). The average growth rates were slightly lower than values reported in the literature for shake flask and bioreactor experiments. For example, reported growth rates for MG1655 range from 1.2 hr^-1^ to 2.0 hr^-1^[57-61]. These variations are due to a number of factors, including variations between physiological behaviour of different strain stocks [54], the exact sampling time during the growth curve [58,60], and particular growth conditions (e.g. pH-controlled bioreactor, shake flask, relative aeration, etc.). The lower growth rates observed in the microtitre plates are consistent with our previous observations [46] and with other literature (e.g. [62]), showing a slight growth rate retardation in microtitre plate assay compared with shake-flask analysis; this is due to mild oxygen limitation [62].

### Expression strength is variable at different loci

**Figure 4:**
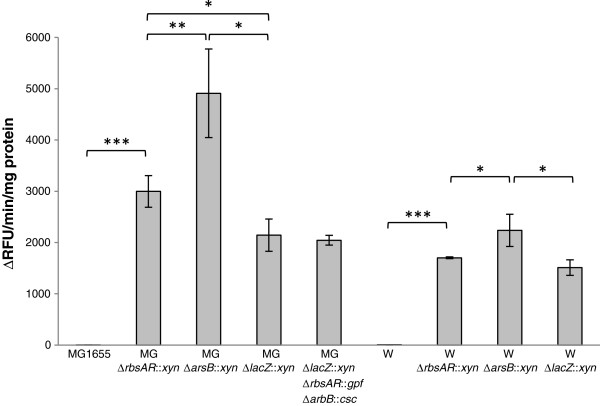
**Xylanase assay.** Xylanase activity was measured in extracts from *E. coli* K-12 (MG1655) and W strains harbouring *xynA* integrated into various different loci. Xylanase is expressed in relative fluorescence units (RFU) per minute per mg protein. Bars represent means; errors are standard deviations (*n* = 3). One-way ANOVA followed by Tukey’s HSD post-hoc analysis was used to resolve differences in means; *, p<0.05, **, p<0.01, ***, p<0.001.

The xylanase insertion cassette was used to assess the difference in expression strength at each locus. Xylanase is used industrially to assist in degradation of plant fibre in the feed, food processing, pulp and paper industries [63]. In addition, xylanase has previously been developed as a reporter gene and its reaction properties have been well characterised [64]. Xylanase activity varied significantly between different integration sites (Figure 4). Xylanase activity also varied between the two strains; however, the relative expression strength between the sites was consistent between strains: xylanase activity was the highest when *xynA* was integrated at the *arsB* locus, followed by *rbsAR* and then *lacZ* (*arsB* >*rbsAR* >*lacZ*). Variation was highest in strain K-12(MG1655), where a 2.3-fold difference was seen between the *arsB* locus and the *lacZ* locus. Expression levels were also higher in K-12(MG1655).

Expression of the xylanase gene should be isolated from the influence of local transcription units due to the presence of transcription terminators [42] in our constructs. However, the proximity of the insertion to the bi-directional origin of replication (*oriC*) also influences gene expression: due to constant genome replication, genes closer to the *oriC* exist at functionally higher copy number [42,65]. Given the locations of our target loci relative to *oriC* (Figure 1), one might therefore expect expression strength to fall in the order *rbsAR* >*arsB* >*lacZ*. However, gene orientation also influences expression levels, with genes on the leading strand being more highly expressed [42]. In *E. coli*, *arsB* and *rbsAR* are encoded on the leading strand, whereas *lacZ* is encoded on the lagging strand. However, in the case of the *xynA* insertions in each locus, the *xynA* gene cassette was always cloned in the opposite orientation relative to the natural expression of the target gene encoding the homologous arms. Thus, *arsB* and *rbsAR* loci *xynA* knock-ins are encoded on the lagging strand, whereas the *lacZ* locus *xynA* knock-in is encoded on the leading strand. Clearly, the expression level advantage enjoyed by genes encoded on the leading strand is insufficient to compensate for relatively greater distance from the *oriC* that *lacZ* is located (Figure 1). However, since the *rbsAR* locus is closer to *oriC* that *arsB*, but *arsB* consistently showed higher expression levels for both strains, other unknown factors must also influence gene expression levels from these loci. In such a small sample size it is not possible to determine what these factors are, and investigation of these phenomena requires further research. It is worth noting that expression of inserted transgenes at loci is relatively predictable [42] as are relative gene expression levels from native genes under a wide variety of conditions [66]. This former observation is supported by the conservation of rank order and relative expression levels between the two strains (Figure 4) and suggests that transgenes integrated at these loci will behave in a predictable fashion.

It should be noted that the actual orientation of the gene being expressed for any given construct made from the KIKO vectors depends on the direction of insertion of the gene cassette between the homologous arms, and this may affect relative expression strength between the loci. It should also be noted that leading strand fidelity is higher than lagging strand fidelity during DNA replication [67]; this, in addition to the generalised expectation of higher expression levels from leading strand-encoded genes [42], suggests that integration in leading strand orientation would be preferable when designing an experiment.

Finally, the higher xylanase activity measured in K-12 relative to W may be due to the relatively higher growth rate of W, which can result in dilution of cellular protein concentrations [68].

### Integration of different exogenous sequences at all three loci in a single strain

**Figure 5:**
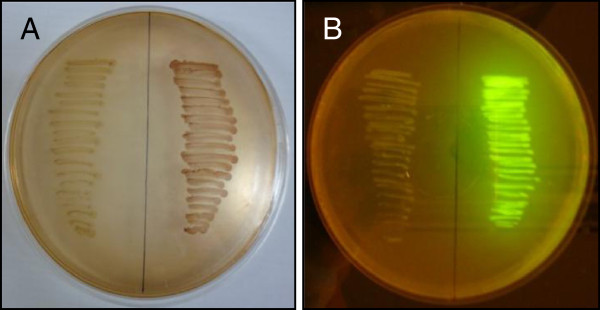
**Sucrose utilisation and GFP activity in strain MGΔ*lacZ*::*xyn *Δ*rbsAR*::*gfp *Δ*arsB*::*csc*. A**) MacConkey agar supplemented with 1% sucrose was used to examine sucrose fermentation in wild type MG1655 (left) and engineered strain MGΔ*lacZ*::*xyn* Δ*rbsAR*::*gfp* Δ*arsB*::*csc*. The ability to utilise sucrose is indicated by a pH change resulting from fermentation products; this is evidenced by red colonies on the MacConkey sucrose plate. **B**) Wild type MG1655 exhibits no fluorescence in the presence of UV light (left), whereas the engineered strain MGΔ*lacZ*::*xyn* Δ*rbsAR*::*gfp* Δ*arsB*::*csc* fluoresces bright green (right).

To demonstrate the functional use of all three integration sites simultaneously, a sucrose metabolism operon encoded by the chromosomally-encoded sucrose catabolism genes (*cscAKB*, 3.9 kb) and green fluorescent protein gene (*gfp*, 0.8 kb) were introduced into the MG1655 strain harbouring the xylanase gene at the *lacZ* locus to produce a triple knock-in strain. Strain K-12(MG1655) cannot normally utilize sucrose [2]. Introduction of the *cscAKB* genes on a high copy number plasmid causes severe phenotypic instability, and we have previously demonstrated that successful sucrose utilization occurs after spontaneous integration of the *csc* genes the *lacZ* locus (this occurs at low frequency in the absence of the λ Red recombinase functions) [46]. While *cscA* alone is sufficient to confer a sucrose-positive phenotype [69-71], *cscAKB* together are required for maximal growth rates on sucrose [72]. Following each integration event the chloramphenicol antibiotic resistance gene was removed via FLP-mediated recombination so that it could be recycled in subsequent rounds of integration. The successful integration of *cscAKB* and *gfp* in strain MGΔ*lacZ*::*xyn* Δ*rbsAR*::*gfp* Δ*arsB*::*csc* was demonstrated by a positive xylanase assay (Figure 4), the ability to ferment sucrose (Figure 5A) and expression of GFP (Figure 5B). The growth rate and xylanase activity in this triple knock-in strain were indistinguishable from the single knock-in strain carrying only the xylanase (Figure 3, Figure 4), indicating that knock-in at all three loci has no negative effect on normal cell growth under these conditions.

It should be noted that iterative rounds of knock-in can result in unintended effects (including deletions and rearrangements) resulting from FRT scars remaining in the genome from previous integration events. In other experiments, we have observed unintended deletions when subsequent secondary DNA constructs are introduced at the same locus [72]. In the case of the KIKO vectors described here, the target loci are well separated in the genome (see Figure 1) and recombination between them would result in large deletions which would presumably be fatal. Strains containing rearrangements and deletions can be excluded from analysis by screening both integration junctions (as described in the Materials and Methods). Use of asymmetric sites for removal of flanking markers, such as λ attL/attR [41,73] or the mutant FRT [74] sites available in *E. coli* or mutant loxP sites [75,76] currently used in eukaryotic systems, would preclude such unintended effects when integrating further constructs at the same locus.

### Comparison of linearised plasmids and PCR products as integration templates

While performing the integration experiments described above using linearised plasmids, we observed a relatively high frequency of single cross-over events, whereby the entire plasmid (instead of just the target region between the homologous arms) was integrated at the target locus. This could be identified by screening at each insertion junction (only a single junction could be amplified by PCR; see Materials and Methods) and was further evidenced by persistent resistance to ampicillin (encoded on the plasmid backbone). The resistance gene cassette could not be cured from these strains because of an inability to select for the pCP20 plasmid [51], which expresses the flippase required to remove sequences between the FRT sites. Moreover, we also observed a high rate of false positives which were resistant to ampicillin but had a wild type locus. This represented substantial background. In order to quantify this background and examine if an alternative approach could be used to decrease background, we performed a direct comparison to investigate the differing efficiencies of integration between linearised plasmids and PCR-amplified fragments (encompassing the two homologous arms and the regions between the arms; see Figure 2A).

The number of colonies obtained for each transformation event was often higher for MG1655 than for W (Table 1), an observation at odds with the competency of the cells: W was an order of magnitude higher in competency than MG1655 (10^9^ CFU/μg plasmid DNA vs. 10^8^ CFU/μg, respectively). We observed this consistently through a variety of other experiments (data not shown), and it means that experiments are generally more facile in MG1655. A generalised dilution of cellular protein (including the recombinase functions) and of transformed DNA fragments as a result of the faster growth rate of W (as described above) may also provide an explanation for the lower transformation efficiency observed. Differences may also be due to variations in the activity of the recombinase functions between the two strains. Regardless, we anticipate that similar variations in transformation and integration efficiency will be seen in other strains.

**Table 1:** Direct comparison of integration using linearised plasmid and pcr products

**Locus**	**Strain**	**Template**	**Colonies**	**WT locus**	**Single X**	**Double X**	**Background**
*arsB*	W	Plasmid	38	33	2	3	92%
		PCR	2	0	2	0	100%
	MG1655	Plasmid	38	4	15	19	50%
		PCR	14	0	2	12	14%
*lacZ*	W	Plasmid	8	5	1	2	75%
		PCR	6	0	1	5	17%
	MG1655	Plasmid	32	25	6	1	97%
		PCR	39	5	12	22	44%
*rbsAR*	W	Plasmid	22	0	16	6	73%
		PCR	7	0	5	2	71%
	MG1655	Plasmid	8	0	8	0	100%
		PCR	15	0	14	1	93%

Templates for each transformation (linearised plasmid or PCR) are noted, as well as the number of colonies obtained from each transformation, the number of colonies having a wild type locus (no integration), the number of colonies having a single cross-over (Single X; integration of entire plasmid) and the number of colonies having a double cross-over (Double X; integration of only the region contained between the homologous arms). Background % is calculated based on the number of WT locus + Single X colonies.

Transformations using linearised plasmid typically produced more colonies than those using PCR product (Table 1). The percentage of false positives and single crossover events was highly variable in the samples examined; however in most cases it was higher in plasmid transformations than in PCR transformations. While the lower number of colonies meant that PCR transformation occasionally did not yield any double crossover events, in most cases the background of single cross-over events was lower in PCR transformations; furthermore, false positives with no insertion at the target locus were very rare. Single cross-overs occurred overall at a similar frequency to double cross-overs across all experiments. Where single cross-over events occurred, the single cross-over consistently occurred with high preference (80–100% across all three loci) on the side homologous with the 3′ sequence of the gene in the insertion locus (data not shown). We have no good explanation for this observation.

The high incidence of false positives (ampicillin resistant colonies with a wild type insertion locus) is surprising. It is unlikely to occur from residual uncut plasmid, because the plasmid replicates via the R6K *ori* and so cannot be maintained in the absence of a λ*pir* gene [48,49]. Therefore, we presume that the plasmid must be integrating elsewhere on the chromosome. Spontaneous mutation is also a possibility, although rates are too high for it to be the only possibility.

There were no clear differences between the different insertion loci, with the exception that false positives were never found when integrations were attempted at the *rbsAR* locus. This may be due to the small sample size used, or may be due to some unknown feature of the *rbsAR* insertion vector/locus.

## Conclusions

Incorporation of large DNA fragments onto the *E. coli* genome at precise, well-defined loci is desirable both for metabolic engineering applications and for examining whole biological pathways. We have constructed a series of knock-in/knock-out (KIKO) plasmid vectors designed to successfully achieve this in one step. We also address the need for multiple reliable insertion sites in one genome in order to facilitate complex engineering in *E. coli* and show that all three loci characterised can be used for integration in a single strain. The variation in expression level at the different loci serves to highlight the need for well characterised integration sites. We have shown the utility of this system in two laboratory strains, and we anticipate that it will be generally applicable across a range of strains.

The KIKO vectors have been used successfully within our laboratory for the integration of a variety of genes for production of industrially relevant products, including the polyhydroxybutyrate operon in *E. coli* W (*phbCBA* + selection marker, 5.8 kb total) and the methylerythritol pyrophosphate isoprenoid pathway in both W and K-12 (8 genes across three loci, including insert sizes of 7.3 kb at the *lacZ* locus and 4.0 kb at the *arsB* locus). Synthetic constructs for expressing multiple genes are typically synthesized commercially unless they are already available. Although the method described here involves linearization of the integration vector, we subsequently found that integration of the entire plasmid can occur at relatively high frequency. This may result from poor integration at the second homologous region, or from integration of residual non-linearised plasmid via host-encoded recombination functions (more likely in wild type unmodified strains that retain their native recombinase functions). We have found that either complete excision of the insert cassette by digestion at both ends (data not shown) or PCR amplification of the insertion cassette (Table 1), followed by gel excision of the fragment (in the former case) and/or DpnI digestion to remove plasmid DNA (in the latter case), significantly increases the frequency of the desired recombination event. We have also used Gibson assembly [77] using the KIKO vectors as PCR templates to successfully construct insertion cassettes for integration. This approach shortens the strain construction process by two days and removes the vagaries of standard cut-and-paste ligation-mediated cloning, and is currently our preferred approach for production of complex insertion cassettes. Although we have not tested them specifically, it is also theoretically possible to use the KIKO vectors as templates to produce knock-out fragments as described by Datsenko & Wanner [23]; the universal priming site P2 is intact, but the P1 site is truncated by two base pairs, necessitating design of a slightly different primer for this application. The KIKO plasmids will be available from Addgene (http://www.addgene.org/).

## Methods

### Bacterial strains, media and growth conditions

Bacterial strains are shown in Table 2 and plasmids are shown in Table 3. R6K-based vectors were maintained in *E. coli* BW23474 [78]. *E. coli* W (NCIMB 8666, National Collection of Industrial, Food and Marine Bacteria, Aberdeen, UK) and MG1655 (*E. coli* Genetic Stock Centre, CGSC 7740) were used as targets for genomic integration. *E. coli* strains were grown in LB medium [79] supplemented with ampicillin (100 μg/mL), kanamycin (50 μg/mL), and/or chloramphenicol (40 μg/mL) as appropriate, unless otherwise stated. Sucrose utilization experiments were performed using MacConkey agar (Difco, BD, North Ryde, NSW, Australia) supplemented with 1% sucrose or M9 medium [79] supplemented with 1 mg/L thiamine and 2% sucrose (M9S).

**Table 2:** Strains used in this study

**Strain**	**Properties**	**Reference/source**
BW23474	Δ*(argF-lac)169,* Δ*uidA4::pir-116, recA1, rpoS396(Am), endA9(del-ins)::FRT, rph-1, hsdR514, rob-1, creC510*	[78]^1^
MG1655	λ-, rph-1	CGSC^1^
MGΔ*arsB*::*xyn*	MG1655 *arsB*::*xynAcatP*; Cm^R^	This study
MGΔ*rbsAR*::*xyn*	MG1655 *rbsAR*::*xynAcatP*; Cm^R^	This study
MGΔ*lacZ*::*xyn*	MG1655 *lacZ*::*xynAcatP*; Cm^R^	This study
MGΔ*lacZ*::*xyn*FRT	MG1655 *lacZ*::*xynA*-FRT; Cm^S^	This study
MGΔ*lacZ*::*xyn* Δ*rbsAR::gfp*-Cm	MG1655 *lacZ*::*xynA-*FRT *rbsAR*::*gfpcatP*; Cm^R^	This study
MGΔ*lacZ*::*xyn* Δ*rbsAR*::*gfp*	MG1655 Δ*lacZ*::*xynA*-FRT Δ*rbsA-rbsR*::*gfp*-FRT; Cm^S^	This study
MGΔ*lacZ*::*xyn* Δ*rbsAR*::*gfp* Δ*arsB*::*csc*-Cm	MG1655 Δ*lacZ*::*xynA*-FRT Δ*rbsA-rbsR*::*gfp*-FRT Δ*arsB*::*cscAKBcatP;* Cm^R^	This study
MGΔ*lacZ*::*xyn* Δ*rbsAR*::*gfp* Δ*arsB*::*csc*	MG1655 Δ*lacZ*::*xynA*-FRT Δ*rbsA-rbsR*::*gfp*-FRT Δ*arsB*::*cscAKB-*FRT; Cm^S^	This study
W	Wild type strain	NCIMB 8666^2^
WΔ*arsB*::*xyn*	W *arsB*::*xynAcatP*; Cm^R^	This study
WΔ*lacZ*::*xyn*	W *lacZ*::*xynAcatP*; Cm^R^	This study
WΔ*rbsAR*::*xyn*	W *rbsAR*::*xynAcatP*; Cm^R^	This study

^1^Purchased from the *Coli Genetic Stock Centre (CGSC)*.

^2^National Collection of Industrial Bacteria, Aberdeen, Scotland. This strain is also archived as ATCC9637 in the American Type Culture Collection.

**Table 3:** Plasmids used in this study

**Plasmid**	**Properties**	**Reference/ source**
pKD3	oriR6Kgamma *bla catP*	[23]
pKD4	oriR6Kgamma *bla aphA*	[23]
pKD46	repA101ts & oriR101 P_araB_*exo*, *bet*, *gam araC bla*	[23]
pCP20	Flp recombinase expression plasmid	[51]
pNPDX2	PBTac2 (Boehringer) carrying modified *xynA* cDNA from *Neocallimastix patriciarum*	[80]
pBAV1K-T5-gfp	pBAV1K-T5 plasmid that carries *gfp* gene	[81]
pCSCX	pCR2.1 plasmid carrying *cscA*, *cscK* and *cscB* gene from *E. coli* W	[46]
pJAS01	Synthetic construct. Contains MCS & FRT-*catP*-FRT cassette flanked by hairpin loops.	This study
p*arsB*-R6K	JSP64/JSP65 PCR product from pKD4 (PvuII; 1,495; R6K *ori* and *bla*) ΩJSP10/JSP11 PCR product (PmeI; 1,109; *arsB’*)	This study
p*lacZ*-R6K	JSP64/JSP65 PCR product from pKD4 (PvuII; 1,495; R6K *ori* and *bla*) ΩJSP57/JSP58 PCR product (PmeI; 1,318; *lacZ’*)	This study
p*rbsAR*-R6K	JSP64/JSP65 PCR product from pKD4 (PvuII; 1,495; R6K *ori* and *bla*) ΩJSP55/JSP56 PCR product (PmeI; 1,034; *rbsAR*’)	This study
pKIKO*arsB*Cm	p*arsB*-R6K (PmeI; 2,604; linear) ΩpJAS01 (PmeI; 1,143; MCS/*catP* cassette)	This study
pKIKO*lacZ*Cm	p*lacZ*-R6K (PmeI; 2,813; linear) ΩpJAS01 (PmeI; 1,143; MCS/*catP* cassette)	This study
pKIKO*rbsAR*Cm	p*rbsAR*-R6K (PmeI; 2,529; linear) ΩpJAS01 (PmeI; 1,143; MCS/*catP* cassette)	This study
pKIKO*arsB*Km	pKIKO*arsB*Cm (XbaI; 2,839; *catP* drop-out) ΩpKD3 (XbaI; 930; *aphA*)	This study
pKIKO*lacZ*Km	pKIKO*lacZ*Cm (XbaI; 2,764; *catP* drop out) ΩpKD3 (XbaI; 930; *aphA*)	This study
pKIKO*rbsAR*Km	pKIKO*rbsAR*Cm (XbaI; 3,111; *catP* drop out) ΩpKD3 (XbaI; 930; *aphA*)	This study
pKIKO*arsB*Ω*xyn*Cm	pKIKO*arsB*Cm (PstI/SalI; 3,763) ΩpNPDX2 (PstI/SalI; 1,108; *xynA*)	This study
pKIKO*lacZ*Ω*xyn*Cm	pKIKO*lacZ*Cm (PstI/SalI; 3,688) ΩpNPDX2 (PstI/SalI; 1,108; *xynA*)	This study
pKIKO*rbsAR*Ω*xyn*Cm	pKIKO*rbsAR*Cm (PstI/SalI; 4,035) ΩpNPDX2 (PstI/SalI; 1,108; *xynA*)	This study
pKIKO*rbsAR*Ω*gfp*Cm	pKIKO*rbsAR*Cm (SacI/SpeI; 4,035) ΩpBAV1K-T5-gfp (*Sac*I/*Spe*I; 1,849; *aphA* &*gfp*)	This study
pKIKO*arsB*Ω*cscAKB*Cm	pKIKO*arsB*Cm (EcoRI; 3,771) ΩpCSCX (EcoRI; 4,064; *cscAKB*)	This study

### Molecular techniques

Standard molecular cloning procedures [79] were employed. Oligonucleotide primers used in this study are described in Table 4. PCR products and restriction endonuclease treated-plasmid DNA were purified using a MinElute PCR purification kit and QIAquick gel extraction kit (Qiagen, Doncaster, VIC, Australia), respectively.

**Table 4:** Primers used in this study

**Primer**	**Application**	**Sequence (5′-3′)**
JSP10	Amplification of *arsB* fragment (PmeI)	GGCGCAGCTGTTTAAACGTCCTGACCATCGTATTGG
JSP11	Amplification of *arsB* fragment (PmeI)	GGGTCAGCTGTTTAAACGTCCCAAATCGCAGCCAAT
JSP22	Screening oligo for KIKO vector inserts	TTCTGCGAAGTGATCTTCCG
JSP55	Amplification of *lacZ* fragment	GCGAGTTTAAACCATTTTCCGTGACGTCTCGT
JSP56	Amplification of *lacZ* fragment (PmeI)	GCGAGTTTAAACAAGACTGTTACCCATCGCGT
JSP57	Amplification of *rbsAR* fragment (PmeI)	GCGAGTTTAAACCAAGCGGCATTGTGTATATC
JSP58	Amplification of *rbsAR* fragment (PmeI)	GCGAGTTTAAACCCCAGTTCATCTTTCGGTTG
JSP64	Amplification of R6K ori and *bla* (PvuII)	TAGGCAGCTGGGAGGATATTCAAATGGACC
JSP65	Amplification of R6K ori and *bla* (PvuII)	CCCCCAGCTGGATGCAGGTGGCACTTTTCG
JSP123	Confirmation of *arsB* genomic insertion	CAACCTGGCTCGACAAAACT
JSP124	Confirmation of *arsB* genomic insertion	GTGTCACAAACAGCACAGGC
JSP125	Confirmation of *rbsAR* genomic insertion	CCGAACTGATGAAAGTGCTC
JSP126	Confirmation of *rbsAR* genomic insertion	GCGTAAATCTAAGCCGAACC
JSP129	Confirmation of *lacZ* genomic insertion	GTCTGAATTTGACCTGAGCG
JSP130	Confirmation of *lacZ* genomic insertion	TCATACAGAACTGGCGATCG
JSP148	Amplification of *xynA* (PstI)	AGGACTGCAGCGGAGCTTATCGACTGCACG
JSP149	Amplification of *xynA* (SalI)	AAACGCTGACTTAACGAGGAGCGGCAGAGG
cscB_F2	Screening oligo for *cscAKB* insertion	ATGGCACTGAATATTCCATTCAGA
KO Test	Confirmation of insertion junction at Cm end	GGAGTGAATACCACGACGAT

Restriction enzyme sites included in primers are underlined, and the enzyme name is listed in the Application column.

### Construction of the KIKO vectors

The KIKO vectors (detailed in Table 5) were constructed in two stages. In the first stage, the R6K origin and ampicillin resistance (*bla*, Ap^R^) gene was amplified from pKD4 [23] using primers JSP64 and JSP65 and ligated to ~ 1 kb fragments amplified from *E. coli* W of *arsB* (primers JSP10 and JSP11), *lacZ* (primers JSP55 and JSP56), and p*rbsAR* (primers JSP57 and JSP58) to generate the base vectors p*arsB*-R6K, p*lacZ*-R6K, p*rbsAR*-R6K respectively. A synthetic cassette that contained a multiple cloning site (MCS) and a chloramphenicol resistance gene (*catP;* Cm^R^) flanked by FRT recombination sites [23] flanked by transcriptional terminators (hairpin loops) from the omega cassette [52] was synthesized in a plasmid backbone (DNA 2.0; CA, USA). In the second round of cloning, the synthetic cassette was excised from the synthesized plasmid using PmeI and subcloned into unique blunt sites within the base vectors (*arsB*, EcoRV; *lacZ*, EcoRV; *rbsAR*, XmnI) to generate the Cm^R^ KIKO vectors pKIKO*arsB*Cm [GenBank KC503965] pKIKO*lacZ*Cm [GenBank KC503966] and pKIKO*rbsAR*Cm [GenBank KC503967]. To generate KIKO vectors conferring kanamycin resistance (*aphA;* Km^R^), the *catP* gene was replaced with an *aphA-*containing fragment from pKD3 [23] using *Xba*I sites. This produced the Km^R^ KIKO vectors pKIKO*arsB*Km [GenBank KC503968], pKIKO*lacZ*Km [GenBank KC503969] and pKIKOKm [GenBank KC503970]. These plasmids will be available from Addgene (http://www.addgene.org/).

**Table 5:** List of kiko vectors, their characteristics and genbank accession numbers

**KIKO vector**	**Target locus**	**Resistance gene**	**GenBank accession**
pKIKO*arsB*Cm	*arsB*	*catP* (Cm^R^)	KC503965
pKIKO*lacZ*Cm	*lacZ*	*catP* (Cm^R^)	KC503966
pKIKO*rbsAR*Cm	*rbsAR*	*catP* (Cm^R^)	KC503967
pKIKO*arsB*Km	*arsB*	*aphA* (Km^R^)	KC503968
pKIKO*lacZ*Km	*lacZ*	*aphA* (Km^R^)	KC503969
pKIKO*rbsAR*Km	*rbsAR*	*aphA* (Km^R^)	KC503970

The resistance gene listed is the gene inserted onto the chromosome after homologous recombination. *Km*^*R*^ kanamycin resistance, *Cm*^*R*^ chloramphenicol resistance.

### Construction of xylanase KIKO vectors

To generate xylanase knock-in constructs, PCR products carrying the xylanase gene (*xynA*) under the control of the strong inducible *tac* promoter were amplified from the plasmid pNPDX2 [63] using the primers JSP148 and JSP149, and cloned into each of the Cm^R^ KIKO vectors using PstI and SalI to generate pKIKO*arsB*Ω*xyn*Cm, pKIKO*lacZ*Ω*xyn*Cm and pKIKO*rbsAR*Ω*xyn*Cm. In these constructs, the *xynA* gene cassette is inserted in the opposite orientation relative to the natural expression of the gene encoding the homologous arms. To generate a *gfp* knock-in construct, the *gfp* gene was digested from pBAV1K-T5-gfp [81] with SacI and SpeI and ligated into pKIKO*rbsAR*Cm at the cognate sites to produce pKIKO*rbsAR*Ω*gfp*Cm. To generate a sucrose utilization gene knock-in construct, the *cscAKB* operon was amplified from pCSCX [46] using primers JSP250 and JSP251. The PCR product was digested with SpeI, purified, and ligated into SpeI-digested pKIKO*arsB*Cm, generating pKIKO*arsB*Ω*cscAKB*. All ligations were transformed into rubidium chloride treated *E. coli* BW23474 chemically competent cells (λ*pir*^+^; see Table 2). For pKIKO*rbsAR*Ω*gfp*Cm, the transformants were plated on LB agar supplemented with chloramphenicol and kanamycin. For pKIKO*arsB*Ω*cscAKB*, the transformants were plated on the LB agar supplemented with chloramphenicol; colonies were inoculated in 1 ml of M9S broth with chloramphenicol and were grown overnight at 37°C to confirm the suc^+^ phenotype. All of the putative recombinants were further screened using colony PCR and confirmed using Sanger sequencing of plasmid DNA. Sequencing was performed by Australian Genome Research Facility (The University of Queensland, St. Lucia, QLD, Australia).

### Strain construction

Engineered strains are shown in Table 2. Gene knock-in was performed by homologous recombination using the arabinose-inducible λ Red recombination system as described previously [23] with minor modifications also described previously [46,72]. KIKO vectors containing xylanase genes were linearized with NotI and 250 ng of purified linear plasmid was electroporated into arabinose-induced W(pKD46) and MG1655(pKD46) electrocompetent cells. In parallel, the integration cassette was also PCR-amplified from each KIKO vector, purified and again 250 ng of product was used to transform cells. The transformation efficiency (competency) of the cells was determined using 120 ng of pACYC184 (New England Biolabs, Ipswich, MA, USA). Integration of the xylanase gene was confirmed by colony PCR using three separate PCRs: 1) a *xynA*-specific primer (JSP148) coupled with a chromosome-specific primer (JSP123 for the *arsB* locus, JSP129 for the *lacZ* locus and JSP125 for the *rbsAR* locus); 2) a *catP*-specific primer (KO test) coupled with a chromosome-specific primer (JSP124 for the *arsB* locus, JSP130 for the *lacZ* locus and JSP126 for the *rbsAR* locus); and 3) a PCR to screen across the whole insertion locus (JSP123 and JSP124, JSP129 and JSP130 and JSP125 and JSP126, respectively). The resulting W-derived strains were designated WΔ*arsB*::*xyn*, WΔ*lacZ*::*xyn*, WΔ*rbsAR*::*xyn*, and the resulting K-12(MG166)-derived strains were designated MGΔ*arsB*::*xyn*, MGΔ*rbsAR*::*xyn*, and MGΔ*lacZ*::*xyn*.

To generate a double knock-in with xylanase and GFP, the Cm^R^ resistance gene was first removed from *E. coli* MGΔ*lacZ*::*xyn* by flippase-mediated recombination as described previously [23,51]. The resulting strain (MGΔ*lacZ*::*xyn*FRT) was transformed with pKD46 and NotI-linearized pKIKO*rbsAR*Ω*gfp*Cm plasmid DNA was integrated into the *rbsA*-*rbsR* locus using the method described above. Transformants were selected on LB agar supplemented with chloramphenicol (25 μg/ml) and kanamycin (25 μg/ml). Integration of the *gfp* gene in the *rbsA*-*rbsR* locus was confirmed by colony PCR using JSP125 and JSP126 primers. Expression of the *gfp* gene in the resulting strain MGΔ*lacZ*::*xyn* Δ*rbsAR::gfp*-Cm was confirmed by colony fluorescence under UV light. The Cm^R^ resistance gene was then eliminated as described above to produce strain MGΔ*lacZ*::*xyn* Δ*rbsAR*::*gfp*. To generate a triple knock-in, the sucrose utilization operon *cscAKB* from NotI-linearized pKIKO*arsB*Ω*cscAKB* was integrated at the *arsB* locus using the method described above. Transformants were selected on LB agar supplemented with 25 μg/ml chloramphenicol and the suc^+^ phenotype confirmed using M9S medium supplemented with 25 μg/ml chloramphenicol. Integration of the *cscAKB* genes was verified by colony PCR using the JSP123 and cscB_F2 primers [72].

### Growth rate analysis

The growth rate of the recombinant strains were determined from 96-well microtitre plate cultures monitored using a FLUOStar Omega multi-detection microplate reader (BMG Labtech, Offenburg, Germany) as described previously [46] with the exception that LB medium was used instead of defined medium.

### Xylanase assay

Strains were cultured in LB to an OD_600_ of 0.6-0.8 then induced for 3 h with 0.1 mM isopropyl-β-D-thiogalactopyranoside (IPTG). The cells were harvested by centrifugation, washed and resuspended in 1 volume of PBS buffer, and lysed with PopCultureTM reagent (Novagen, Billerica, USA). The lysed cell culture was clarified by centrifugation and the xylanase activity measured using an EnzChek® Ultra Xylanase assay kit (Invitrogen, Eugene, USA). Fluorescence was measured on FLUOStar Omega multi-detection microplate reader (BMG Labtech, Offenburg, Germany) every 30 sec min for 30 min at room temperature using excitation at 360 nm and emission detection at 460 nm. Commercial xylanase from *Thermomyces lanuginosus* (Sigma, St Louis, USA) was used to develop a standard curve. Protein concentration was determined by the method of Bradford [82] using a BioRad Protein Assay kit (BioRad, Gladesville, Australia) and bovine serum albumin as a standard. Xylanase activity was expressed as change in relative fluorescence over time, adjusted for total protein present (ΔRFU/min/mg protein).

### Statistical analysis

The xylanase activity and the growth rate data were analyzed using analysis of variance (ANOVA). All statistical analyses were performed using R software (http://www.r-project.org/).

## Abbreviations

csc: Chromosomally-encoded sucrose catabolism; CmR: Chloramphenicol resistance; FRT: Flippase recombinase target sites; GFP: Green fluorescent protein; HA: Homologous arm; KIKO: Knock-in/knock-out; KmR: Kanamycin resistance; MCS: Multiple cloning site; QC: Quality control; RFU: Relative fluorescence units; xynA: Xylanase gene.

## Competing interests

The authors declare that they have no competing interests.

## Authors’ contributions

This study was designed by JAS, CEV, LKN and MB. Plasmid vector generation, strain generation and strain characterization was performed by JAS and SS. The manuscript was written by CEV, JAS, and SS and approved by all authors prior to submission.
